# Reviews

**Published:** 1864-09

**Authors:** 


### REVIEWS.

*A Treatise on the Chronic Inflammation and Displacements of the Unimpregnated Uterus. By* WM. H. BYFORD, A. M., M. D., *Professor of Obstetrics, etc., etc., Chicago Medical College, Medical Department Lind University. Philadelphia:* LINDSAY & BLAKISTON—1864.

This is a carefully written practical monograph upon the inflammatory diseases of the uterus; in every chapter of which may be seen evidence that the author has not only a theoretical but also a practical familiarity with the subject upon which he writes. He speaks positively from experience, and rejects what he has found worthless, retaining, for the instruction of others, whatever he has found valuable and true.

The first chapter is devoted to general considerations of the subject of uterine diseases, and a brief rehearsal of the various opinions which have been and still are entertained of the character and influence upon the general system of local uterine disease or displacement. Chapter second is upon the sympathetic accompaniments of uterine disease, in which are described the general symptoms which affect the system, and the affections of the stomach, bowels, liver, nervous system, circulatory and respiratory functions, with mental and moral derangements, etc., etc.

In chapter third are portrayed with great truthfulness and completeness the local symptoms which indicate uterine inflammation.

Chapter four is devoted to the etiology of this disease; sexual indulgence, reading improper books, cold, constipation, standing, abdominal supporters, pessaries, severe exercise, hemorrhoids, pregnancy, abortions, especially from violence, bad management after abortions, labor, decomposing substances in tne vagina or uterus after labor, or at other times, vaginities of infancy and childhood, gonorrhœa, etc., etc., are regarded as the principal causes of uterine inflammation.

Chapter fifth is upon Prognosis; sixth, complications of the disease; seventh, position of the inflammation; eighth, its progress and termination; ninth, contains careful consideration of the means of diagnosis; tenth, eleventh and twelfth are upon the proper means of cure—the general and local treatment of the disease; thirteenth, is upon treatment of submucous inflammation; fourteenth, displacements, their philosophy and treatment.

To this is appended a report of interesting and important cases. Together it constitutes one of the most plain, practical and valuable works upon this department of medical practice, and we most earnestly recommend all our readers to supply themselves with this volume. We believe that it is correct and truthful, and accords with the best and most established views.

---

*Lectures on Orthopædic Surgery; delivered at the Brooklyn Medical and Surgical Institute, with numerous illustrations, By* Louis Bauer, M. D., M. R. C. S., *Eng.; Professor of Anatomy and Clinical Surgery; Licentiate of the New York State Medical Society; Member of the New York Pathological Society, of the American Medical Association; Corresponding Fellow of the London Medical Society; Health Officer of the City of Brooklyn, etc. Re-printed from the Philadelphia Medical and Surgical Reporter. Phiadelphia:* Lindsay & Blakiston, 1864.

This work furnishes us with, first, historical sketch of Orthopædic Surgery, definitions, boundaries, general causes, etc., etc. Second, a complete and highly rational consideration of the various forms of taliepes, and the most efficient methods of relief or cure. Deformities of the hip and knee joints are described with great minuteness and truthfulness, and the most available means of rectifying mal-positions, are described in detail, and with great care. The consideration of diseases and deformities of the spine is also upon the same plan. The work is fully illustrated, so that the opinions of the author and of many other observers may be fully understood. The instruments found useful in the treatment of all the different deformities are represented, and nothing is omitted necessary to give a complete understanding of the various means and appliances necessary in correcting the various mal-positions of the body. There are but few works upon this subject in this country worthy of confidence, and this monograph will be particularly acceptable to those surgeons who attempt the treatment of such deformities. It is claimed that this department of surgery is entitled to a distinct specialty. The great difficulty in securing proper care in such cases unless they are placed in charge of experienced attendants, or better, if in institutions devoted exclusively to care of such patients contributes to the necessity of "specialty" in this branch; yet every surgeon should understand the principles and best manner of treatment, and should not neglect cases which come under his care, and cannot be otherwise provided for. It cannot be doubted that much indifference is manifested by general practitioners, and deformities which would be obviated without difficulty are neglected, or postponed until hopelessly incurable. If this work is carefully perused it will not only instruct, but will also stimulate to a better treatment of this class of deformities.

---

*Lectures on Medical Education, or on the proper method of Studying Medicine.* By SAMUEL CHEW, M. D., *Professor of the Principles and Practice of Medicine and of Clinical Medicine in the University of Medicine. Philadelphia:* LINDSAY & BLAKISTON, 1864.

This book is written with great good taste, and shows the author to have an elevated and cultivated mind, which he is devoting to the good of the medical profession. The following brief extract from the preface will show the objects and character of the work. Medical students should *all read it, and read all of it:*

"I have lately commenced the study of Medicine, and write to consult you on that occasion. What books ought I to read? How shall I read them to most advantage? How many hours daily should be devoted to reading? Is much reading necessary? I am acquainted with several physicians, who, I think, have read but little. I know one who declares that his only guide in the treatment of diseases is his own experience, and that he has never opened a book since he graduated; his friends are of opinion that his reading had been equally extensive before that epoch; and yet he has been extremely successful in business, and has much practice and great reputation. On what branches ought I to attend Lectures during the first session? Is it advisable to take Notes of the Lectures? Is it necessary to attend Clinical Lectures in a Hospital? If necessary, why is it so when we have so many books on the Practice of Medicine? Are Dissections necessary in the study of Anatomy? They must be very disagreeable; would not plates answer in their stead? Is it necessary to pay attention to Medical Auscultation? My old preceptor considers it wholly useless, and says that he can investigate diseases of the chest better without that mode of examination than any one else can with the help of a cart-load of Stethescopes. What is the general character of our Medical Schools? Does the Medical Profession in this country stand as high in public estimation at present, as it did in former times? Has it not lost something of its ancient reputation and *prestige?*"

Such are some of the inquiries which almost every student of Medicine addresses to those of whom he seeks counsel in relation to the conduct of his professional studies. It is in reply to these and similar questions that the present volume has been prepared. In it are imbodied the opinions and advice respecting certain subjects connected with Medical Education, which the author has long been in the habit of giving to his classes of pupils."

